# Assessing the utility of the tipping point ratio to monitor HIV treatment programmes in the era of universal access to ART

**DOI:** 10.1016/j.idm.2018.03.005

**Published:** 2018-03-14

**Authors:** Simon de Montigny, Marie-Claude Boily, Benoît R. Mâsse, Kate M. Mitchell, Dobromir T. Dimitrov

**Affiliations:** a CHU Sainte-Justine Research Center, Montreal, QC, Canada; b School of Public Health, University of Montreal, Montreal, QC, Canada; c Department of Infectious Disease Epidemiology, Imperial College, London, United Kingdom; d Fred Hutchinson Cancer Research Center, Seattle, WA, United States

**Keywords:** HIV, Mathematical modeling, Antiretroviral treatment, Tipping point

## Abstract

**Background:**

The epidemiological tipping point ratio (TPR) has been suggested as a useful indicator to monitor the scale-up of antiretroviral treatment (ART) programmes and determine when scale-up is sufficient to control the epidemic. TPR has been defined as the ratio of yearly number of new HIV infections to the yearly number of new ART initiations or to the yearly net increase in the number of people on ART. It has been used to rank the progress of treatment programmes across countries, with the objective of reaching a TPR value under 1. Our study aims to assess if TPR alone can be used as an indicator of ART success across settings by comparing the expected changes in HIV incidence and ART coverage when TPR is maintained constant over time. In particular, we focus on the effect of ART initiation timing (emphasis on ART being initiated early or late during HIV progression) on the interpretation of the TPR.

**Methods:**

We used a dynamic model of HIV transmission in South Africa representing ART rollout leading to universal treatment in 2017. The model is calibrated to HIV incidence, HIV prevalence and ART coverage in 2012 in South Africa, and 1000 simulations are selected for the base-case scenario. To measure the effect of TPR, we simulate TPR-preserving interventions, maintaining TPR (yearly number of new ART initiations denominator) at the value observed in 2019 (between 0.65 and 1.25) for 15 years. We compare ART coverage and HIV incidence across TPR values and across strategies in which ART access is prioritized differently. In a secondary analysis, we illustrate the sensitivity of new ART initiations to ART retention, and we compare both definitions of the TPR.

**Results:**

Our analysis shows that HIV incidence reduction is weakly correlated to TPR: the same reduction in HIV incidence (15%) can be achieved by implementing the same strategy with a wide range of TPR maintained (0.65–1.12). Assuming high retention in ART, TPR-preserving strategies prioritizing early ART initiation yield greater reduction in HIV incidence than strategies where most individuals initiate ART late. High ART coverage is associated with low HIV incidence and it can be reached with a TPR below or equal to one with strategies favoring early ART initiation. Low ART retention over time results in higher HIV incidence even if TPR is maintained low. If ART retention is low, strategies prioritizing late ART initiation are associated with lower HIV incidence than strategies where ART is initiated early. Maintaining a fixed TPR value based on the net increase in people on ART gives higher HIV incidence reduction and requires fast ART scale-up.

**Conclusion:**

Our analysis suggests that the TPR is not an adequate indicator of ART programme impact, without information on ART coverage and retention. Achieving early initiation and adherence to treatment to improve ART coverage might be as important as attaining a specific TPR target. Comparisons of TPR in different settings should account for differences in epidemic conditions.

## 1 Introduction

The epidemiological tipping point ratio (TPR) has been defined as the ratio of the number of annual new HIV infections to the number of annual new antiretroviral treatment (ART) initiations or to the annual net increase in the number of people on ART. This statistic has been suggested as a useful indicator to monitor the scale-up of ART programmes and determine when a programme is likely to have reached a level sufficient to successfully control the HIV epidemic (US PEPFAR, 2012). Intuitively, reducing the TPR below 1 appears desirable as it implies that more infected individuals initiate treatment than become infected. Therefore, fewer infected individuals should remain untreated over time, increasing coverage. Proponents of TPR as an indicator of ART progress say that TPR values maintained less than 1 show “a country getting ahead of its epidemic” (US PEPFAR, 2012). Still, to control the epidemic, the timing of ART initiation might be as important as the number of HIV+ persons initiating treatment due to the increased opportunities for HIV transmission prior to ART initiation at a later stage of infection.

One problem with the applicability of the TPR as an independent metric comes from the fact that alternative definitions are used in the literature with respect to how the denominator of the ratio is estimated. Even though first mentions of the TPR (US PEPFAR, 2012) suggest to use the net increase of in the number of people on ART as denominator, many research publications and even some PEPFAR country reports use the denominator defined or approximated by the number of new ART initiations, number of new people placed on ART, new people initiating or enrolled in ART or becoming suppressed (Coggin, 2014; Granich et al., 2015; Hall, Espinoza, Harris, Tang, & Mermin, 2015; Henry, 2015; ONE, 2016; PAHO, 2013; Tanzania Third National Multi-Sectorial Strategic Framework for HIV and AIDS, 2013; US PEPFAR, 2015). This definition has been widely used which is the reason we use it in our main analysis (we refer to this definition as the TPR). We refer to the definition using the net ART increase as the “net TPR” and investigate its performance in a secondary analysis. Different versions of these two definitions have been used interchangeably even within single documents which often creates confusion and the false sense that they have the same meaning.

There is no clear recommendation on how to use the TPR in monitoring ART programmes. It has been suggested that the TPR should only be derived once ART coverage exceeds 67% (Bass, de Lacy Donaldson, Fisher, & Warren, 2014). It also has been pointed out that if ART coverage gets very high, the TPR may be uninformative with few HIV-infected persons not already on treatment (US PEPFAR, 2013). Thus, once 67% ART coverage is achieved, the TPR can be used to track progress towards the targets of 90% and 95% coverage (90-90-90 and 95-95-95 WHO initiatives). However, the 67% threshold for TPR interpretation has been disregarded in some TPR rankings of countries or US states (Granich et al., 2015; Hall et al., 2015). It remains an open question if TPR values are comparable between settings with different trends in the timing of ART initiation. Moreover, ART retention, a key factor for effective ART scale-up, is not reflected by the TPR using ART initiation as denominator.

Mathematical models are invaluable tools in evaluating the effectiveness of biomedical interventions for HIV prevention (Dimitrov et al., 2010, 2015; Hallett et al., 2011; Vickerman et al., 2006; van de Vijver et al., 2013). In this study, we use a mathematical model to assess the validity of the TPR as an indicator of an ART programme's progress. The model reproduces the dynamics of HIV transmission in South Africa and represents ART rollout starting in 2002 with progressive expansion of eligibility criteria (based on CD4 count) until universal treatment is introduced in 2017.

In our main analysis, we simulate different treatment strategies, introduced in 2020 to maintain the TPR at the value observed in 2019. These strategies have different ART access priorities based on CD4 count categories (>200, 200–350, 350–500 and >500). Maintaining the TPR constant over a prolonged period of time enables us to clearly see how the TPR relates to HIV incidence without the relationship being obscured by changes in TPR over time. Our objective is to verify if lower TPR values and if particular strategies are associated with larger reduction in HIV incidence and greater increase in ART coverage. This analysis informs the validity of the TPR as a measure of ART progress by investigating the importance of lowering the TPR value and helps determine the merit in ranking ART progress in different settings by TPR value only. We emphasize the importance of the distribution of ART initiations across different HIV-infected subgroups and demonstrate that it deserves more attention when ART programmes are planned or implemented.

## 2 Methods

### 2.1 Model description

**Fig. 1:**
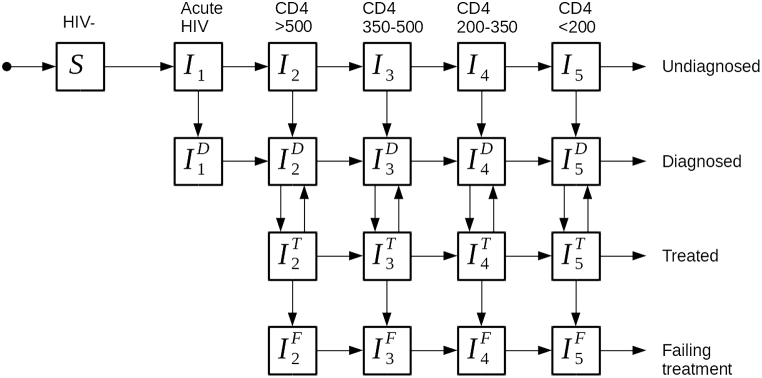
Compartmental flow diagram of HIV transmission model.

We used a dynamic compartmental model of HIV transmission representative of the HIV epidemic among the sexually active population (15–49 years old) in South Africa in the era of ART rollout from the year 2002 onwards (see Fig. 1, see also Section 1 of Supplementary information). The model was originally developed to estimate the effectiveness of future intervention with multi-dose HIV vaccines (de Montigny et al., 2018).

Individuals can either be HIV- or HIV+, and progression towards AIDS is characterized by passage through one acute phase and four subsequent chronic phases distinguished by CD4 count (CD4 >500, CD4 350–500, CD4 200–350 and CD4 <200 cells/mm^3^).

HIV transmission is based on a fixed probability of infection per sexual act in a sero-discordant partnership. Individuals in acute and CD4 <200 phases are characterized by a higher probability of transmitting HIV. Individuals change sexual partners at a fixed rate, and perform a fixed number of sexual acts per year, a fraction of which are protected by condoms.

### 2.2 Treatment

As infected individuals are diagnosed, they can initiate treatment depending on ART eligibility (following treatment guidelines in South Africa): CD4 <200 eligibility starting in 2002, partial CD4 200–350 eligibility added in 2010, full CD4 <350 eligibility added in 2012, CD4 350–500 eligibility added in 2015, CD4 >500 eligibility added in 2017 (see Table 1). We assume that individuals in acute HIV compartments move to CD4 >500 before entering treatment due to the very short duration of the acute phase. Those initiating treatment immediately benefit from a 73%–99% reduction of HIV transmission risk (Cohen et al., 2011) accounting for imperfect viral suppression of some ART users. The range of possible values allows for different levels of adherence to ART in simulations.

**Table 1:** **Key model parameters.** See Supplementary information for more details.

Parameter	Value or calibration range	Justification
HIV transmission risk reduction on ART	73%–99%	Cohen et al., 2011
Mean duration of acute HIV phase (years)	0.21	Wawer et al., 2005 Todd et al., 2007 Lodi et al., 2011
Mean duration of CD4 >500 phase (years)	1.12	
Mean duration of CD4 350–500 phase (years)	3.70	
Mean duration of CD4 200–350 phase (years)	4.20	
Mean duration of CD4 <200 phase (years)	2.95	
Base-case ART initiation rate in CD4 <200 phase (person years) - 2002 to 2009	0.8–1	Reflects ART eligibility progression in South Africa.
Base-case ART initiation rate in CD4 <200 phase (person years) - in 2010 and after	1.5–2	
Base-case ART initiation rate, in CD4 200–350 phase (person years) - in 2010 and 2011	0.2–0.3	
Base-case ART initiation rate in (person years) - CD4 200–350 phase in 2012 and after - CD4 350–500 phase in 2015 and after - CD4 >500 phase in 2017 and after	0.6–0.8	

On ART, progress towards AIDS is slowed with the mean duration of each phase extended by 100%–200%. Also, treatment dropout or failure can occur. Individuals dropping out can later re-initiate ART, but individuals failing treatment cannot do so.

### 2.3 Parameters and calibration

Parameters related to the natural history of HIV (durations of HIV phases, probability of HIV transmission by phase) were informed from published studies (Lodi et al., 2011; Todd et al., 2007; Wawer et al., 2005). Other parameters have been calibrated to uncertainty intervals for population size, HIV prevalence, HIV incidence, proportion of HIV+ adults on treatment, and proportion of HIV+ adults who are undiagnosed from the South African National HIV Prevalence, Incidence and Behavior Survey, 2012 (Shisana et al., 2014) as follows. Parameters are generated randomly, and the model is simulated in the period 2002–2012. In 2012, if each of the five statistics lies in its uncertainty interval, the parameter set is selected for the base-case scenario of ART expansion. This process is repeated until 1000 parameter set are selected, which reflect the variety of epidemic conditions that are compatible with the calibration statistics. See Supplementary Fig. S1 illustrating the HIV incidence, HIV prevalence, ART coverage and adult population curves based on the simulations with selected parameter sets for the 2002–2017 period, and see Sections 4, 5 of Supplementary information and Tables S1–S10 for more details.

### 2.4 Simulation scenarios

**Fig. 2:**
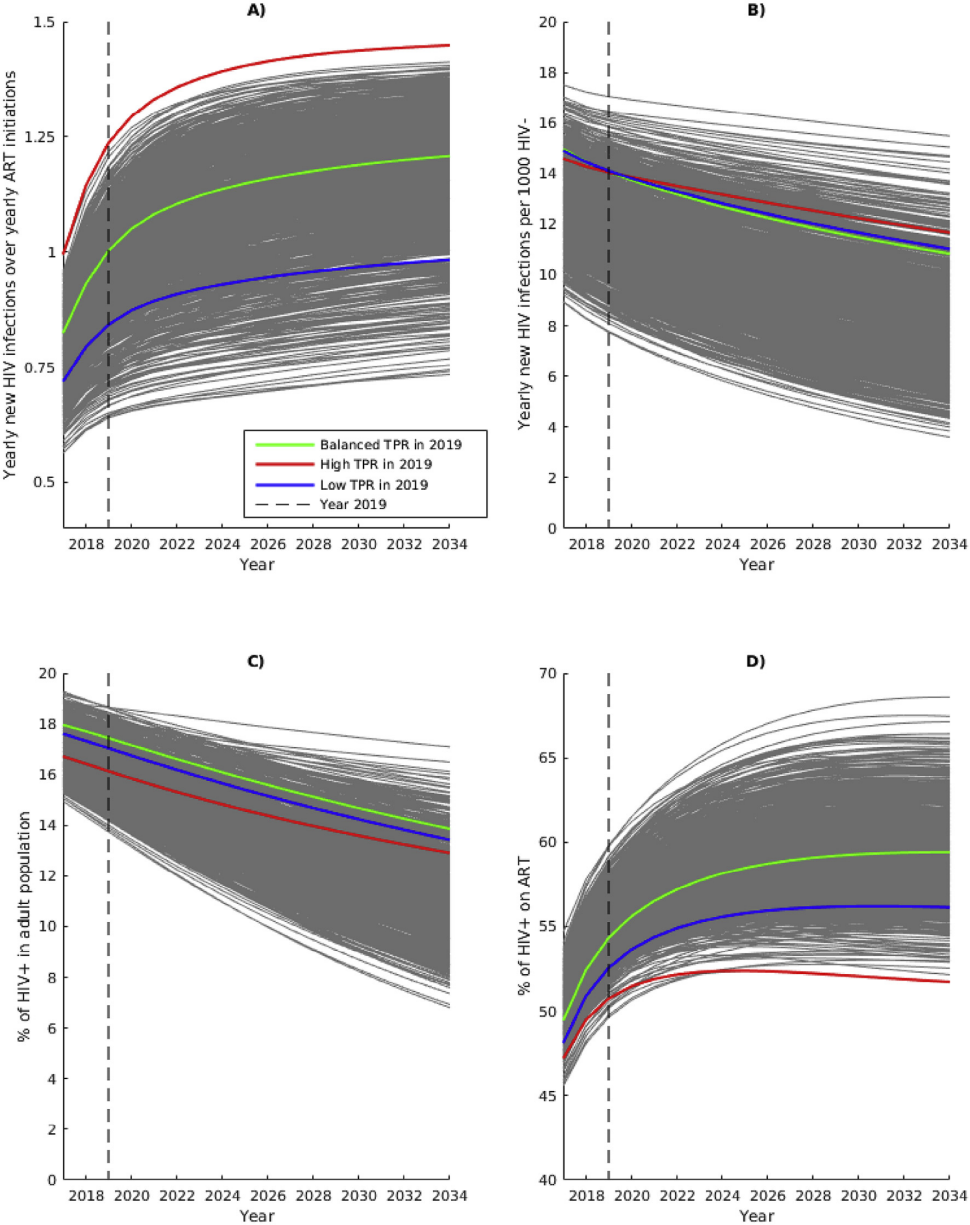
**Base-case simulations.** Temporal dynamics of A) TPR value; B) HIV incidence; C) HIV prevalence and D) ART coverage up to 2034, using the 1000 calibrated simulations, assuming that the ART initiation rates remain unchanged. Highlighted simulations with high TPR (red), balanced TPR (green) and low TPR (blue) have similar HIV incidence in 2019 and are used in the comparison of the impact of TPR-preserving strategies.

In the base-case scenario, all 1000 simulations using calibrated parameter sets (we refer to them as calibrated simulations) are extended until 2034, assuming that the ART initiation rates remain unchanged. In these calibrated simulations, the TPR is rising over the 15-year period from 2019 to 2034. Three of the base-case simulations (see Fig. 2) with similar HIV incidence but different TPR (low, balanced and high) at the start of the intervention in 2019 are selected for our main analysis, which compares HIV incidence after 15 years of different intervention strategies across TPR values.

In the intervention scenarios, TPR-preserving strategies are implemented starting in 2020. To study the link between the TPR and HIV incidence, we increase ART initiation rates to keep the TPR constant over time and measure the effect on incidence of doing so. Maintaining the TPR constant helps highlight this effect that could be obscured by changes in TPR over time.

We study different TPR-preserving strategies by increasing the number of ART initiations sufficiently to maintain the TPR value at the level of 2019 (implemented by varying the rates of ART initiation in different CD4 groups). The “CD4<XX first” strategy assumes that all new ART initiations are distributed proportionally between all HIV-positive untreated compartments with CD4<XX, including both diagnosed and undiagnosed. If not sufficient, they are supplemented with individuals from the adjacent CD4 groups. The “CD4<XX priority” strategy assumes that base-case yearly ART initiation rates by CD4 group are maintained. The rest of the ART initiations needed are distributed proportionally between all diagnosed untreated compartments with CD4<XX. If not sufficient, they are supplemented with undiagnosed individuals with CD4<XX individuals followed by infected individuals from the next CD4 groups. We developed those two strategies using CD4 <200, CD4 <350, CD4 <500 and universal (all CD4) thresholds as restriction criteria, yielding a total of eight strategies described in more detail in Section 7 of Supplementary information and Table S11.

These strategies yield higher ART initiation rates than the base-case rates in the first years after the intervention. Over time, as incidence declines, so do the ART initiation rates, thus keeping the TPR constant. Relative to the base case, more HIV+ individuals are treated yearly just after the intervention, but later on, fewer people may be treated yearly, depending on the epidemic parameters of the simulation. Note that this does not entail a decline of ART coverage if few HIV+ individuals drop out of treatment.

Motivated by the fact that an HIV cure is not yet available, we assume that each newly infected individual remains infected for life. In contrast, after initiating ART, the treated individuals do not necessarily remain on ART indefinitely. Therefore, we simulate scenarios with different rates of ART dropout to explore the importance of ART retention to the reported results.

### 2.5 Outcomes of interest

We estimated the effective TPR value (the ratio of the yearly number of new HIV infections over yearly number of new ART initiations), the HIV incidence (number of yearly new HIV infections per 1000 HIV- persons) and the ART coverage (percent ratio of HIV+ adults on ART to HIV+ adults) for the base-case scenario and the intervention scenarios in which TPR is maintained constant by different TPR-preserving strategies. We also estimated the relative reduction in HIV incidence $\left(100\%-100\%\frac{\text{HIV}\;\text{incidence}\;\text{with}\;\text{TPR}-\text{preserving}\;\text{strategy}}{\text{HIV}\;\text{incidence}\;\text{in}\;\text{base}-\text{case}\;\text{scenario}}\right)$ and the distribution of ART initiations by CD4 stage (yearly number of HIV+ individuals from CD4<XX group initiating ART over yearly number of HIV+ individuals initiating ART). All metrics are estimated over the 2020–2034 period.

To evaluate the importance of TPR for the dynamics of the HIV epidemic, we analyzed the correlation between the TPR value maintained at a constant level and the reduction in HIV incidence in 2034 for the eight TPR-preserving strategies that we designed. To facilitate the comparison of different strategies, we have selected three specific simulations (one with low TPR, one with balanced TPR near 1, one with high TPR), each having the same HIV incidence. We compared the incidence value in 2034 across all strategies for these three simulations. We also highlighted the crucial role of ART coverage in the relation between TPR and HIV incidence.

## 3 Results

### 3.1 Base-case simulations

To provide context for estimating impact of TPR-preserving interventions, we first project the evolution of the HIV epidemic over the 2017–2034 period, assuming base case ART initiation rates by CD4 groups are maintained.

Fig. 2 illustrates the dynamics of TPR value, HIV incidence, HIV prevalence and ART coverage over the period 2017–2034 for 1000 base-case simulations. Overall, without a TPR-preserving strategy, the TPR is rising over time (median 0.78, range 0.56–1.00 in 2017 to median 1.17, range 0.73–1.45 in 2034). HIV incidence is declining (median 12.8, range 8.9–17.5 in 2017 to median 8.4, range 3.6–15.5 in 2034). HIV prevalence is also decreasing from median 17% (range 15–19%) in 2019 to median 11% (range 8–18%) in 2034. ART coverage is initially rising fast after introducing the universal access to ART in 2017 but then stabilizes over time. Overall, no simulation reaches the recommended TPR interpretation threshold of 67% ART coverage by 2019 (a few reach that threshold in the second half of the 2017–2034 period).

**Fig. 3:**
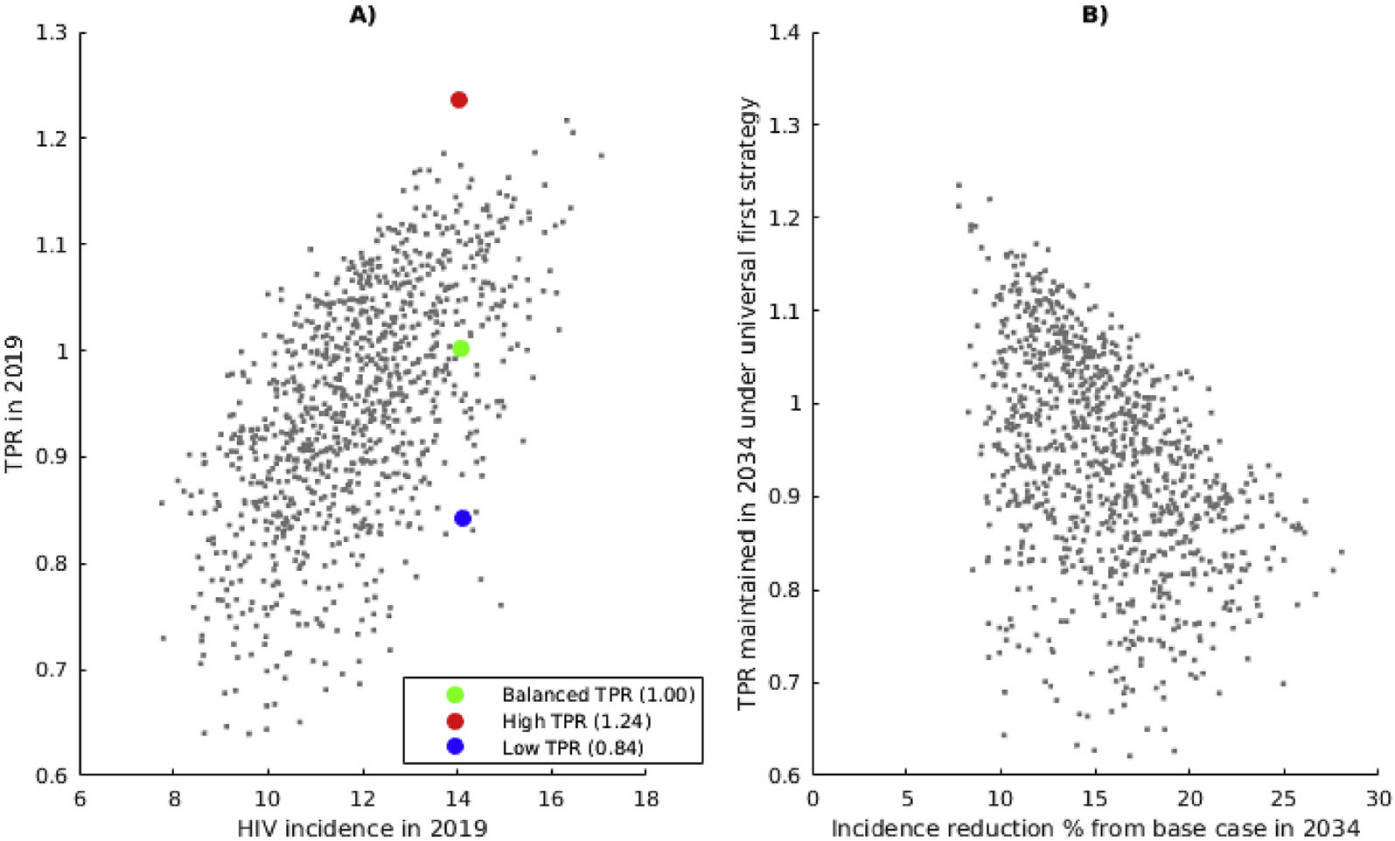
**Correlation between TPR and HIV incidence.** Scatterplot of A) TPR vs HIV incidence at the start of the intervention in 2019 (data derived from Fig. 2A and B) and B) TPR vs. HIV incidence reduction achieved at the end of the intervention in 2034, assuming that the “universal first” strategy is implemented (Pearson's r = −0.43). Highlighted simulations with high TPR (red), balanced TPR (green) and low TPR (blue) have similar HIV incidence in 2019 and are used in the comparison of the impact of TPR-preserving strategies.

Notably, a wide range of HIV incidence values are observed for the same TPR value. For instance, a balanced TPR of 1 may correspond to HIV incidence between 9 and 16 infections per 1000 person-years (Fig. 3A). Similarly, the same HIV incidence can be observed in combination with different TPR values. For instance, HIV incidence of 14 infections per 1000 person-years comes with TPR ranging from 0.84 to 1.24 (Fig. 3A). We further analyze the impact of different TPR-preserving strategies on the HIV epidemic using three simulations with similar HIV incidence (14 new HIV infections per 1000 HIV- per year) and different TPR (highlighted in Fig. 2): one with high TPR in 2019 (TPR = 1.24), one with balanced TPR (TPR = 1.00) and one with low TPR (TPR = 0.84).

### 3.2 Impact of fixed TPR-preserving strategy across settings with different TPR

Intuitively, we expect that keeping the TPR low will lead to lower HIV incidence. Therefore, it is expected that if the same TPR-preserving strategy is implemented in different settings, then larger HIV incidence reduction will be achieved with low TPR than with balanced and high TPR. We have simulated all 1000 epidemic conditions, assuming that TPR is maintained constant and assuming equal access to ART by all infected individuals (“universal first” strategy), and compared the expected reduction in HIV incidence to base-case simulations (Fig. 3B). Overall, our analysis suggests that larger reductions in incidence are expected if a lower TPR is maintained. However, any fixed value of TPR is associated with a wide range of levels of HIV incidence reduction, depending on the epidemic conditions (Fig. 3B). Incidence-reduction ranges associated with different TPR values often overlap. As a result, 20% reduction in incidence can be expected with TPR maintained around 1.05 in some simulations while reduction in incidence below 10% is expected with TPR maintained around 0.7 under different conditions. Similar results are obtained for other TPR-preserving strategies (see Supplementary Fig. S2).

**Fig. 4:**
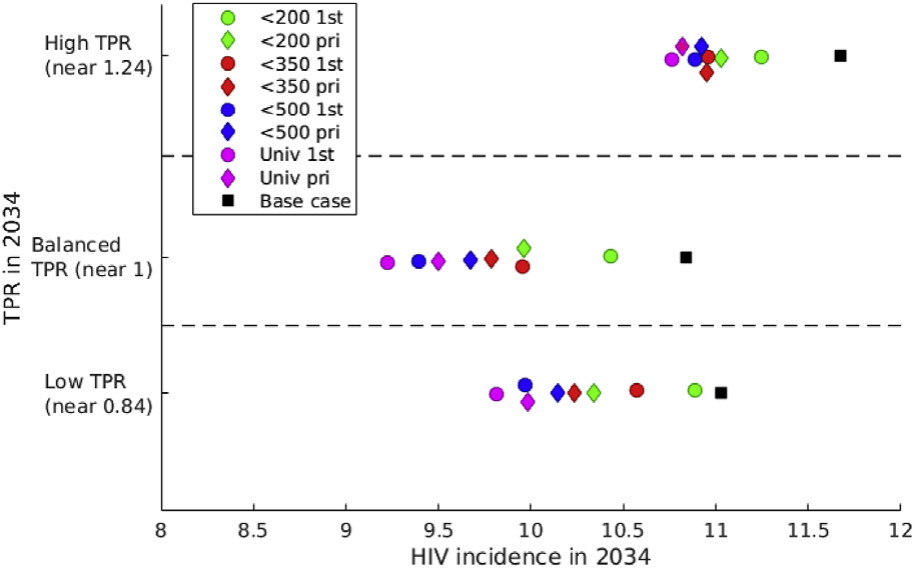
**Effect of TPR-preserving strategies on HIV incidence.** Expected HIV incidence in 2034. “First” strategies are noted “1st” and “priority” strategies are noted “pri”. HIV incidence in 2019 is 14 new infections per 1000 person-years for all these simulations.

In the three settings selected with similar HIV incidence in 2019 and different TPR, we also compared the expected HIV incidence achieved in 2034 with different TPR-preserving strategies (Fig. 4). Comparison of simulations under the same strategy (same color and shape) shows that the lowest HIV incidence is consistently reached in settings with balanced TPR which does not support the use of TPR for ranking ART programmes.

A supplementary analysis was performed using the base-case simulation with balanced TPR in 2019 and applying all ”CD4<XX first” strategies maintaining TPR at 0.9, 1 or 1.1 from 2020 to 2034. Comparison of HIV incidence curves under different interventions, presented in Supplementary Fig. S3, supports the conclusions of our main analysis that the TPR by itself is not enough to quantify the impact of the ART programme.

### 3.3 Comparison of HIV incidence across TPR-preserving strategies

In Fig. 4, the uncertainty in HIV incidence, for a given TPR value maintained, is substantial in comparison to the uncertainty in HIV incidence across different TPR values. For instance, if the number of new ART initiations have been balanced with new infections over 15 years (balanced TPR), the expected HIV incidence varies between 9.2 and 10.5 infections per 1000 person-years, depending on how new ART initiations are distributed between different CD4 groups (different strategies). It stands out that the worst strategy is the one concentrating new ART initiations in the CD4 <200 group.

Overall, interventions with early ART initiation (i.e. at high CD4 count, >350 or >500; blue and magenta in Fig. 4) are expected to have larger impact than intervention with late ART initiation (at lower CD4 count, <200 or <350; green and red in Fig. 4). Note that “priority” strategies (diamonds) correspond to better incidence reduction than “first” strategies (circles) for low CD4 thresholds (<200, <350) while the opposite relation is observed for high CD4 thresholds (<500, universal). These relations between early and late ART initiation and between “priority” and “first” strategies can also be observed over all 1000 calibrated simulations in Supplementary Fig. S2. The distributions of new ART initiations by CD4 stage during different interventions are described in Tables S12–S20.

### 3.4 ART coverage

**Fig. 5:**
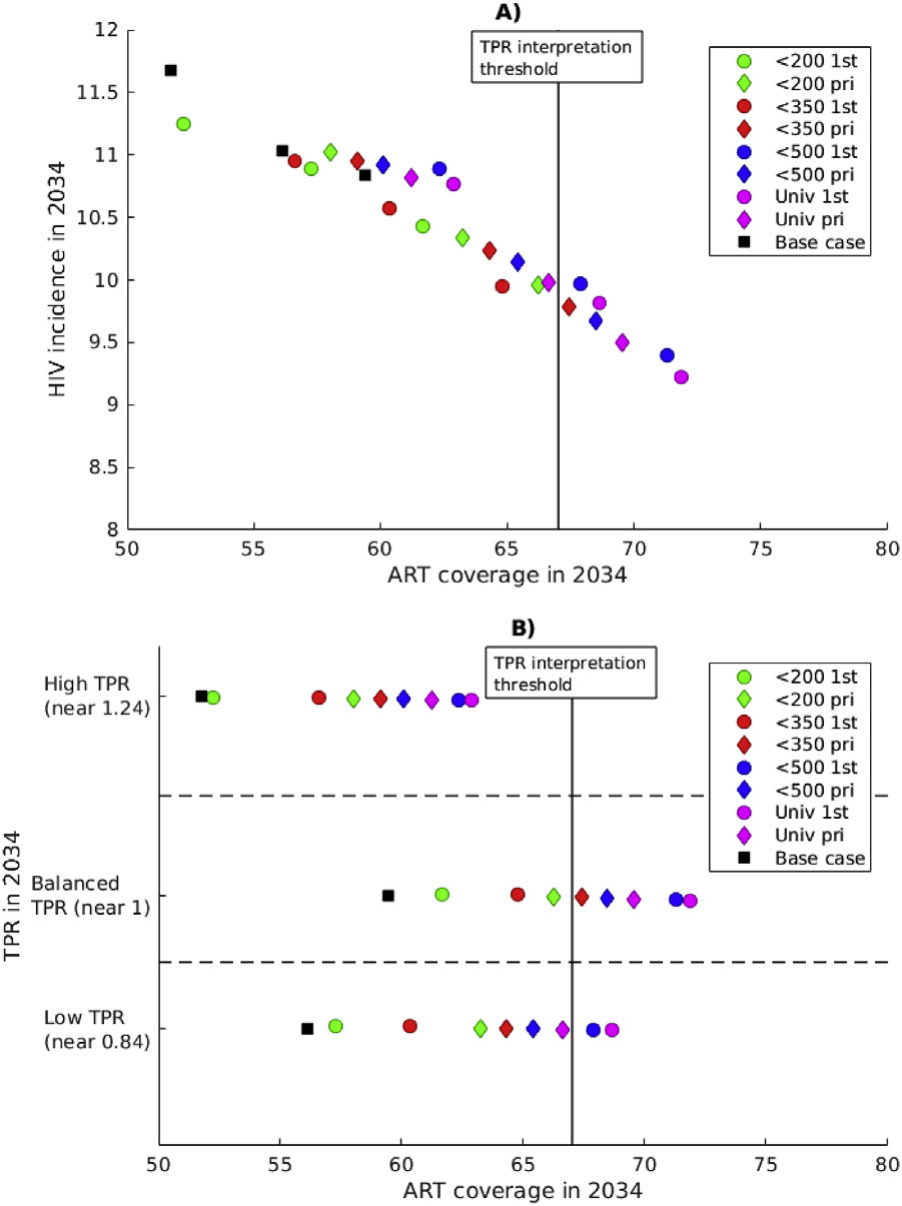
**Effect of TPR-preserving strategies on ART coverage.** A) Effect of ART coverage on HIV incidence. **B)** Expected ART coverage in 2034 for different TPR-preserving strategies.

Next, we analyze the ART coverage under different ART expansion strategies preserving different TPR values (see Fig. 5). It is clear that the expected ART coverage is a very good predictor of the expected HIV incidence (Fig. 5A). Interventions with early ART initiation (higher CD4 threshold) yield better coverage. For each TPR value, the order of the simulated strategies by ART coverage is the same and follows a similar order as levels of HIV incidence discussed above (Fig. 5B). Notice that better ART coverage is achieved in the setting with balanced TPR. Also, the “CD4 <200 first” strategy leads to substantially lower ART coverage than all other strategies. This, to a large extent, explains the results reported in Fig. 4. Finally, some of the strategies with high CD4 threshold (CD4 <500 and universal) are projected to reach the TPR interpretation threshold of 67% ART coverage in 2034. Revisiting Fig. 4, we conclude that even when ART coverage is higher than 67%, incidence reduction cannot be ranked by TPR. Clearly, ART coverage is a better predictor of HIV incidence reduction than TPR across scenarios.

### 3.5 What if ART retention is poor?

In the analysis to this point, we have assumed that fewer than 10% of treated individuals drop out of ART annually. However, this assumption may have a critical influence on the presented results as it favors strategies with early ART initiations in which people will remain on treatment for a very long time. Therefore, we simulated exploratory scenarios with high ART dropout to illustrate an additional difficulty in the TPR interpretation (Table 2). We ran these scenarios in the epidemic setting having the lowest TPR in 2019 (0.64) where HIV incidence in 2019 is 8.2 per 1000 person-years and where ART coverage rises over the 67% TPR interpretation threshold in two years. In this setup, we increased ART dropout, starting in 2027 when ART coverage is 70.2%, to the values 0.1, 0.25 and 0.5, corresponding to 10-, 4- and 2-year average duration on ART, respectively. Table 2 shows the effect of these modifications on HIV incidence, ART coverage and TPR in 2034 for all “CD4<XX first” strategies compared to the unmodified simulation. The effect of this modification on the temporal dynamics of the epidemic is illustrated in Supplementary Fig. S4.

**Table 2:** Effect of augmentation of ART dropout rates under “CD4<XX first” strategies on TPR, HIV incidence and ART coverage in 2034 in epidemic setting with low TPR (0.64) maintained.

Strategy	ART dropout rates modification in 2027			
	Main analysis	Rates = 0.1	Rates = 0.25	Rates = 0.5
CD4<200 first	0.62	0.63	0.64	0.64
	4.2	5.0	7.3	9.5
	62.6%	55.6%	40.4%	31.0%
CD4<350 first	0.62	0.63	0.64	0.64
	3.9	4.9	7.5	9.9
	67.8%	60.8%	45.7%	34.3%
CD4<500 first	0.62	0.63	0.64	0.64
	3.7	5.0	7.7	10.3
	74.1%	64.9%	48.1%	35.5%
Universal first	0.62	0.63	0.64	0.64
	3.7	4.9	7.7	10.2
	74.6%	65.3%	48.3%	35.6%
Cell format (2034 metrics)	TPR value			
	HIV incidence			
	ART coverage			

Not surprisingly, high ART dropout leads to lower ART coverage and substantially higher HIV incidence. In these scenarios, individuals move to ART to match the 2019 TPR target, but they do not remain on treatment for long enough to effectively reduce HIV incidence via the protective effect of ART. As a result, the low TPR is not indicative of ART success. Note that with the increase in dropout, the relative impact of the strategies with late ART initiation improves compared to those with early ART initiation. For instance, under the main analysis with high retention to ART, the lowest HIV incidence (3.7 per 1000 person-years) is achieved with early ART initiation (“universal first”) while the highest HIV incidence (4.2 per 1000 person-years) is expected when low CD4 groups have preferential access to ART (“CD4 <200 first”). In contrast, if the annual ART dropout rate is increased to 25%, the lowest HIV incidence (7.3 per 1000 person-years) is achieved when low CD4 groups have preferential access to ART (“CD4 <200 first”) while the highest HIV incidence (7.7 per 1000 person-years) is expected with early ART initiation (“universal first”). Clearly, when people do not stay on ART long, it is most beneficial for the population to target those with the highest infectiousness (CD4 <200).

Supplementary Fig. S4 shows that TPR spikes immediately after the revision of the ART retention rate in 2027, but afterwards, it returns towards its target value of 0.64. During this spike, the TPR remains under 1. This demonstrates that, in theory, an increasing epidemic with decreasing ART coverage over time can be observed even if the TPR is maintained under 1.

### 3.6 Comparison between TPR definitions

We compared the results obtained in the main analysis with another definition of the TPR proposed and used in the literature (see Section 6 of Supplementary information). In Granich et al. (2015), the number of new ART initiations is estimated by the difference of cumulative number of individuals on ART in consecutive years. Using that TPR denominator to define a “net TPR” (it counts the net increase in number of individuals on ART), we modified our TPR-preserving interventions to require an additional number of ART initiations before counting the new ART initiations necessary to maintain the TPR. This additional number compensates the number of individuals dropping out of ART or dying on treatment in the previous year.

We implemented the “universal first” TPR-preserving strategy on the calibrated simulations aiming to preserve a fixed net TPR value over time. The targeted net TPR in this secondary analysis was selected randomly between 0.6 and 1.8. Our analysis showed that maintaining net TPR values often requires very fast ART scale-up which results in ART coverage near 100% after 5 years. Therefore we focused our attention on the 5-year period after the start of the TPR-preserving strategy (2020–2024). We measured the resulting HIV incidence reduction (relatively to base case) in 2024 and the effective average TPR values derived from projected simulations over 2020–2024 under both definitions (TPR and net TPR). We averaged the TPR to account for fluctuations induced by the compensatory mechanism (see Supplementary information, Fig. S5).

**Fig. 6:**
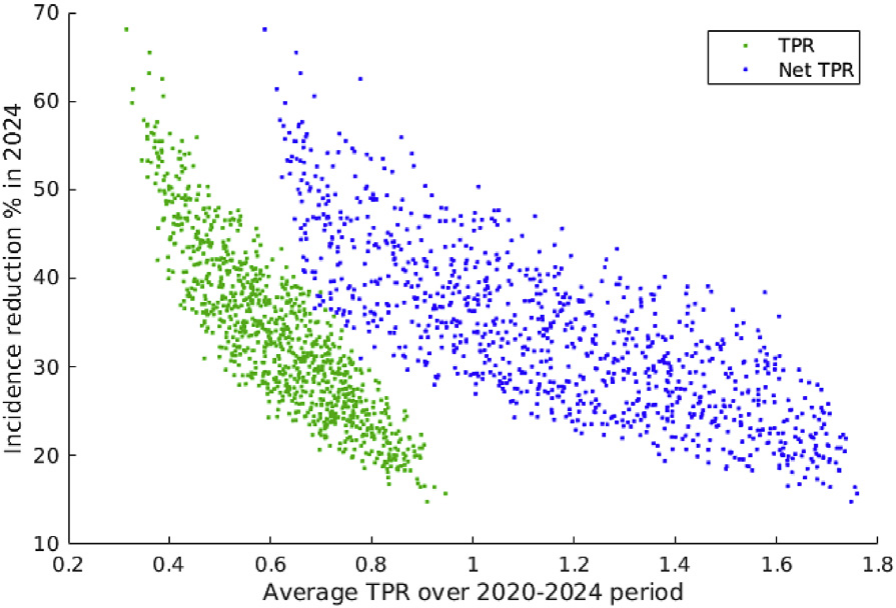
**Comparison between TPR definitions.** Scatterplot of average TPR vs HIV incidence in 2024, assuming that the “universal first” strategy is implemented. Green dots represent results using the definition from the main analysis while blue dots represent results using the alternative definition (net TPR) using net ART increase as denominator.)

Results shown in Fig. 6 demonstrate the substantial difference between both TPR definitions. What is common is that the same HIV incidence reduction (35%) can be achieved with a wide range of TPR (TPR 0.42–0.72, net TPR 0.68–1.58). TPR which counts ART compensation as new initiations yields lower incidence reduction for the same value as net TPR, e.g. TPR = 0.8 corresponds to 19–30% incidence reduction while net TPR = 0.8 corresponds to 29–54%. The difference between early and late ART initiation is small when net TPR is used due to compensation of ART dropout (see Supplementary information, Fig. S6).

## 4 Discussion

Following the usage of the TPR in the literature, it may be incorrectly assumed that reaching and maintaining a TPR value under 1 is a critical milestone in the expansion of ART, with lower values having greater impact on the epidemic. Our analysis demonstrated that this interpretation is not accurate for the TPR (with new ART initiations for the denominator). We simulated interventions in which ART initiation is adjusted to follow a TPR target value in order to characterize the effect of the TPR on HIV incidence reduction, which is among the key goals of ART scale-up strategies such as US National HIV/AIDS Strategy and UNAIDS 90-90-90. We showed that the measure of ART success in terms of HIV incidence reduction only marginally depends on the TPR. The analysis of selected simulations with the same HIV incidence in 2019 shows that larger HIV incidence reduction can be achieved with balanced TPR near 1 compared with low TPR (near 0.84), even when ART coverage is over the proposed 67% interpretation threshold. Similar results are observed in simulated HIV epidemics having the same HIV incidence but distinct epidemic conditions. Our study suggests that epidemic conditions and programme performance (e.g. ART retention) must be taken into account before interpreting the TPR. Thus, comparisons between TPR values across settings should be avoided without additional analyses. Furthermore, the complexity of the interplay between the HIV epidemic and ART scale-up warrants further analysis and modeling to appreciate whether ART programmes are heading towards success or failure.

In places where the TPR is maintained constant over time, if HIV incidence decreases, then the rates of new treatment initiations must go down as well. However, this does not necessary lead to lower ART coverage since the coverage level depends on how long HIV-infected patients remain on ART. Our analysis demonstrated that ART coverage can increase even with ART initiation rates going down as long as treated individuals are retained in care for a long time. This suggests that ART retention is more important to the impact of the ART programme than the TPR value itself.

We found that the effect of TPR on HIV incidence depends on programme conditions (TPR-preserving strategies) and epidemic conditions (1000 calibrated simulations). Based on this study, we conclude the TPR would be inadequate as a sole indicator of the success of ART programmes. We demonstrated that the distribution of new ART initiations should be taken into account to understand or predict the long-term trend of the HIV epidemic in a given setting. Strategies of ART scale-up where a substantial proportion of individuals with CD4 >500 or CD4 350-500 initiate treatment are necessary to achieve high ART coverage within 15 years, assuming that ART dropout is kept to a minimum. In this case, our analysis suggests that ART coverage is a better predictor of programme success than the TPR. Therefore, interventions that measure progress in terms of the overall proportion of virally suppressed among those infected with HIV will trace more precisely their impact on the HIV epidemic. One example is the 90-90-90 initiative of UNAIDS which, if achieved, will increase the proportion of all HIV infected individuals who are virally suppressed to 73%.

It is unlikely that HIV elimination (less than 1 new infection per 1000 HIV- per year) will be achieved by 2034 if a substantial increase in ART coverage is not reached quickly (none of our simulations reach that target). We found that high ART coverage is associated with low HIV incidence. Thus, very low TPR values resulting in very high ART coverage would be needed to progress towards this objective, with adequate monitoring of ART coverage to validate progress. This result is in line with other recent modeling studies (Cori et al., 2014; Eaton et al., 2012) which found that reaching this target is unlikely in the short term. This differs greatly from Granich, Gilks, Dye, De Cock, and Williams (2009), which uses very optimistic treatment scenarios.

We found confusion in the literature around the computation of the TPR denominator. The “increase in new patients on ART” (US PEPFAR, 2013) or “number of persons who initiate ART” (Hall et al., 2015) cannot be negative. However, estimating that number by the difference of cumulative number of individuals on ART in consecutive years (Granich et al., 2015) or cumulative number of individuals achieving viral load suppression (Hall et al., 2015) can yield negative numbers, due to treatment dropout or failure. In Hall et al. (2015), it is claimed that a negative denominator corresponds to a TPR above 1 when in fact this TPR value should be negative. In our main analysis, the denominator is the number of HIV+ persons entering treatment, which is always positive. In our analysis of net TPR with ART losses compensated, we used the net increase in individuals on ART for the denominator. The choice of definition to be used had a very large impact on model results and on TPR interpretation. Note that in our simulations where the parameter for HIV transmission risk reduction on ART is close to 99%, initiating ART is almost equivalent to achieving viral load suppression. In particular, this parameter had a 98.6% value in the analysis of poor ART retention.

Our model and its results are comparable to other treatment-as-prevention models, except for our focus on the TPR. Our work stands out as almost no details are available in the literature about this indicator, its validity and its long term effects on the epidemic trends.

Our model has several limitations. It is restricted to the South African epidemic in the era of ART, so our analysis could fail to apply to dissimilar settings. To simplify calibration, the model is not stratified by sex, which presupposes that the flow of infection from men to women is equal to that from women to men (May, Anderson, & McLean, 1988). The current version of the model is calibrated using South African National HIV Prevalence, Incidence and Behavior Survey data from 2012. The new 2016 survey (not out yet) is expected to provide additional information on HIV prevalence, incidence and treatment cascade in South Africa which may require re-calibration of the model. Our base-case simulations depend on current trends of ART initiation with the decision of South Africa to move towards universal ART. The effect of this change in access is not currently known. The assumption about progression through HIV phases while on treatment may underestimate the extension of life for individuals with CD4 <200, which might not be representative of current health outcomes in South Africa. Finally, many factors are excluded from our model such as heterogeneity in sexual risk behavior and geographical distribution of HIV in South Africa.

In light of our findings, we plan to pursue further analysis of the TPR along two lines of investigation. First, to determine the significance, if any, of the notion of a “tipping point,” we will study the relation between the TPR and the basic reproduction rate in a simplified model. Second, we will proceed with an analysis of TPR trends in scenarios of ART scale-up towards 90-90-90/95-95-95 targets to determine the usefulness of the TPR for measuring ART progress in specific epidemic settings.

## 5 Conclusion

In HIV surveillance, the TPR alone is not sufficient to indicate programme impact without further information on ART coverage and the timing of ART initiation modulated by the distribution of ART initiations across CD4 stages. Our results support the idea that reaching a high ART coverage level is required, but not sufficient by itself, for interpreting the TPR correctly due to the importance of adherence to treatment. We noted that there is confusion in the literature about the definition of the TPR. In a supplementary analysis, we showed that TPR interpretation can be very sensitive to this definition.

Our analysis suggests that TPR alone should not be used to compare ART programmes across countries. A careful assessment of epidemic conditions, ART coverage and ART dropout should accompany TPR rankings. Promoting early initiation and adherence to treatment to achieve high ART coverage might be as important as demonstrating that a specific TPR target has been met.

With the recent recognition that UNAIDS objectives for 2020 are going off track (UNAIDS, 2016), ART programme monitoring is of utmost importance. Further modeling efforts are needed to properly inform ART programmes. Tracking ART coverage is essential to validate ART scale-up strategies. Our results call for caution in using an indicator that is not thoroughly understood, and further work is required to better assess the TPR and determine its appropriate usage.

## Appendix A Supplementary data

mmc1 mmc1
