# Supplementary material for: Assessing the utility of the tipping point ratio to monitor HIV treatment programmes in the era of universal access to ART
Source: Infect Dis Model. 2018 Mar 14;3:85–96. doi: 10.1016/j.idm.2018.03.005 (PMC6326263; doi:10.1016/j.idm.2018.03.005)
Supplement: mmc1 [file mmc1.docx]

Supplementary information

Assessing the utility of the tipping point ratio to monitor HIV treatment programmes in the era of universal access to ART
Simon de Montigny, Marie-Claude Boily, Benoît R. Mâsse, Kate M. Mitchell, Dobromir T. Dimitrov

**1. Epidemic model description**

We developed a deterministic compartmental model to study the tipping point ratio, an epidemiological indicator of ART programme success. We simulate the HIV epidemic in South Africa in the era of ART rollout, focusing on the transmission dynamics in the sexually active population (15-49 years old).

The modeled population is composed of susceptible individuals ($S$) and infected individuals ($I$), see Fig.1. Infected individuals are stratified by HIV progression in five stages (1: acute HIV, 2: CD4 >500, 3: CD4 350-500, 4: CD4 200-350, 5: CD4 <200) and by treatment status (undiagnosed, $D$: diagnosed, $T$: treated, $F$: failing treatment). See Fig.1 for the compartmental flow diagram of the model and Table 1 and Table S1-S10 for the parameter values used in the analysis.

**2. Model equations**

$$N=S+\sum_{k=1}^{5} \left( I_{k}+I_{k}^{D} \right)+\sum_{k=2}^{5} \left( I_{k}^{T}+I_{k}^{F} \right)$$

$$\frac{d}{dt}S=\pi N-\left( \lambda+\mu\right)S$$

$$\frac{d}{dt}I_{1}=\lambda S-\left( \nu_{1}+\delta_{1}+\mu\right)I_{1}$$

$$\frac{d}{dt}I_{k}=\nu_{k-1}I_{k-1}-\left( \nu_{k}+\delta_{k}+\mu\right)I_{k} ;k=2, 3, 4, 5$$

$$\frac{d}{dt}I_{1}^{D}=\delta_{1}I_{1}-\left( \nu_{1}^{D}+\mu\right)I_{1}^{D}$$

$$\frac{d}{dt}I_{k}^{D}=\delta_{k}I_{k}+\delta_{k}^{T}I_{k}^{T}-\left( \nu_{2}^{D}+\gamma_{2}^{D}+\mu\right)I_{k}^{D} ;k=2, 3, 4, 5$$

$$\frac{d}{dt}I_{2}^{T}=\gamma_{2}^{D}I_{2}^{D}-\left( \nu_{2}^{T}+\delta_{2}^{T}+\theta_{2}^{T}+\mu\right)I_{2}^{T}$$

$$\frac{d}{dt}I_{k}^{T}=\nu_{k-1}^{T}I_{k-1}^{T}+\gamma_{k}^{D}I_{k}^{D}-\left( \nu_{k}^{T}+\delta_{k}^{T}+\theta_{k}^{T}+\mu\right)I_{k}^{T} ;k=3, 4, 5$$

$$\frac{d}{dt}I_{2}^{F}=\theta_{2}^{T}I_{2}^{T}-\left( \nu_{2}^{F}+\mu\right)I_{2}^{F}$$

$$\frac{d}{dt}I_{k}^{F}=\nu_{k-1}^{F}I_{k-1}^{F}+\theta_{k}^{T}I_{k}^{T}-\left( \nu_{k}^{F}+\mu\right)I_{k}^{F} ;k=3, 4, 5$$

**3. Force of infection**

$$\lambda=\frac{\rho}{N}\left( \sum_{k=1}^{5} \left( R_{k}I_{k}+R_{k}^{D}I_{k}^{D} \right)+\sum_{k=2}^{5} \left( R_{k}^{T}I_{k}^{T}+R_{k}^{F}I_{k}^{F} \right) \right)$$

$$R_{k}=1-\left( 1-\left( 1-\alpha_{c} \right)b_{k} \right)^{\frac{cn}{\rho}}\left( 1-b_{k} \right)^{\frac{(1-c)n}{\rho}}$$

$$R_{k}^{D}=R_{k}^{F}=R_{k}$$

$$R_{k}^{T}=\left( 1-\varepsilon_{k} \right)R_{k}$$

**4. Parameters for base-case simulations**

Parameters are grouped thematically in Tables S1-S7. Each table contains either **fixed** or **calibrated** parameters: each parameter is either set to a fixed value, or it is generated randomly (following a uniform distribution in a given range) and subjected to a calibration procedure (see Section 5).

| **Parameter** | **Description** | **Range** | **Notes** |
| --- | --- | --- | --- |
| $\pi$ | Recruitment rate | 0.04-0.045 | $x_{1}$ and $x_{2}$ random in range 0-1. $\pi=0.04+0.005\cdot\max\left( x_{1},x_{2} \right)$. $\mu=0.02+0.005\cdot\min\left( x_{1},x_{2} \right)$.  Ranges informed from (1). |
| $\mu$ | Exit rate | 0.02-0.025 |  |

**Table S1 - Demographic parameters (calibrated).**

| **Parameter** | **Description** | **Value** | **Notes** |
| --- | --- | --- | --- |
| $1/\nu_{1}$ | Acute phase duration  HIV+ undiagnosed | 0.21 | Acute phase duration informed from (2).  Median durations from (3, 4) adjusted to obtain mean duration.  Parameter $\nu_{5}$ is AIDS death rate. |
| $1/\nu_{2}$ | CD4 >500 duration  HIV+ undiagnosed | 1.12 |  |
| $1/\nu_{3}$ | CD4 350-500 duration  HIV+ undiagnosed | 3.70 |  |
| $1/\nu_{4}$ | CD4 200-350 duration  HIV+ undiagnosed | 4.20 |  |
| $1/\nu_{5}$ | CD4 <200 duration  HIV+ undiagnosed | 2.95 |  |
| $1/\nu_{k}^{D}$ | HIV phase duration  HIV+ diagnosed | $1/\nu_{k}$ | As for HIV+ undiagnosed |
| $1/\nu_{k}^{F}$ | HIV phase duration  HIV+ failing treatment | $1/\nu_{k}$ | As for HIV+ undiagnosed |

**Table S2 - Duration of HIV phases (fixed).**

| **Parameter** | **Description** | **Range** | **Notes** |
| --- | --- | --- | --- |
| $\nu_{k}^{T}$ | 1 / phase duration  HIV+ on treatment | $\frac{1}{3}\nu_{k}$- $\frac{1}{2}\nu_{k}$ | $x_{1}$ random in range 0-1. $\nu_{k}^{T}=\left( \frac{1}{3}+\frac{1}{6}x_{1} \right)\nu_{k}$  (same multiplier for each $\nu_{k}$). |

**Table S3 - Duration of HIV phases (calibrated).**

| **Parameter** | **Description** | **Range** | **Notes** |
| --- | --- | --- | --- |
| $\delta_{k}$ | Rate of HIV diagnosis  CD4 >200 | 0.1-0.15 | Random (same value for $k=1, 2, 3, 4$) |
| $\delta_{5}$ | Rate of HIV diagnosis  CD4 <200 | 0.2-0.4 | Random  (faster diagnosis due to onset of AIDS) |
| $\gamma_{5}^{D}$ | Rate of ART initiation  CD4 <200 | 0.8-2 | Random in range 0.8-1 (2002 to 2009)  Random in range 1.5-2 (2010 and after) |
| $\gamma_{4}^{D}$ | Rate of ART initiation  CD4 200-350 | 0-0.8 | Equal to 0 (2002 to 2010)  Random in range 0.2-0.3 (2010 to 2011)  Random in range 0.6-0.8 (2012 and after) |
| $\gamma_{3}^{D}$ | Rate of ART initiation  CD4 350-500 | 0-0.8 | Equal to 0 (2002 to 2014)  Equal to $\gamma_{4}^{D}$ (2015 and after) |
| $\gamma_{2}^{D}$ | Rate of ART initiation  CD4 >500 | 0-0.8 | Equal to 0 (2002 to 2016)  Equal to $\gamma_{4}^{D}$ (2017 and after) |

**Table S4 - Diagnostic and ART initiation parameters (calibrated).**

| **Parameter** | **Description** | **Range** | **Notes** |
| --- | --- | --- | --- |
| $\delta_{k}^{T}$ | Dropout rate | 0.01-0.1 | Random  ($\delta_{2}^{T}\leq\delta_{3}^{T}\leq\delta_{4}^{T}\leq\delta_{5}^{T}$) |
| $\theta_{k}^{T}$ | Failure rate | 0.01-0.1 | Random  ($\delta_{2}^{T}\leq\delta_{3}^{T}\leq\delta_{4}^{T}\leq\delta_{5}^{T}$) |
| $\varepsilon_{k}^{T}$ | HIV transmission risk reduction | 0.73-0.99 | Random in range informed by (5)  (same value for $k=2, 3, 4, 5$) |

**Table S5 - ART adherence and retention parameters (calibrated).**

| **Parameter** | **Description** | **Value** | **Notes** |
| --- | --- | --- | --- |
| $b_{1}$ | Infection probability  Acute HIV partner | 0.055 | Values adjusted to obtain a distribution of infection flow by HIV phase comparable to other models in (6). |
| $b_{2}$ | Infection probability  CD4 >500 partner | 0.0021 |  |
| $b_{3}$ | Infection probability  CD4 350-500 partner | 0.0006 |  |
| $b_{4}$ | Infection probability  CD4 200-350 partner | 0.0011 |  |
| $b_{5}$ | Infection probability  CD4 <200 partner | 0.0033 |  |

**Table S6 - Infection probability per sex act between serodiscordant partners (fixed).**

| **Parameter** | **Description** | **Range** | **Notes** |
| --- | --- | --- | --- |
| $\rho$ | Rate of partnership acquisition | 1-1.75 | Random |
| $n$ | Yearly number of sex acts | 80-120 | Random integer  ($n/\rho$ : yearly number of sex acts per partnership) |
| $c$ | Fraction of protected sex acts | 0.1-0.4 | Range informed from (7) |
| $\alpha_{c}$ | Protection efficacy per sex act | 0.7-1 | Range informed from (8) |

**Table S7 - Sexual behavior parameters (calibrated).**

**5. Model initialization and calibration**

The compartments $S$ and $I_{k}$ are initialized following data described in Table S8. The progression of ART eligibility in South Africa is described in Table S9. We assume that before ART rollout in 2002, there is a negligible fraction of individuals on ART such that the compartments $I_{k}^{T}$ and $I_{k}^{F}$ are initially empty. We further assume the compartments $I_{k}^{D}$ are empty such that, following the onset of ART rollout, linking to care through a clinical evaluation is required before treatment can be initiated.

| **Initialization data** | **Range** |
| --- | --- |
| Adult population size  (millions) | 23.959-25.441 |
| HIV prevalence  (% of HIV+ in population) | 13.9%-17.6% |
| % of HIV+ in acute HIV phase | 1.6% |
| % of HIV+ in CD4 >500 phase | 44.4% |
| % of HIV+ in CD4 350-500 phase | 25.6% |
| % of HIV+ in CD4 200-350 phase | 18.9% |
| % of HIV+ in CD4 <200 phase | 9.5% |

**Table S8 – Model initialization data for 2002 (9, 10)**

| **Years** | 2002 to 2009 | 2010, 2011 | 2012 to 2014 | 2015, 2016 | 2017 and after |
| --- | --- | --- | --- | --- | --- |
| **ART eligibility** | CD4 <200 | CD4 <350* | CD4 <350 | CD4 <500 | Universal ART |

**Table S9 - ART eligibility in South Africa by year.
* CD4 200-350 restricted eligibility.**

The model is calibrated using a Monte-Carlo filtering scheme. Parameters of tables S1, S3, S4, S5 and S7 are generated randomly. Using this set of parameter values, the model is simulated in the 2002-2012 period. Simulation statistics are calculated (see Table S10) and compared to target ranges informed by the 2012 South African National HIV Prevalence, Incidence and Behaviour Survey (9). If a set of values gives rise to statistics matching all target ranges, it is kept in a list of calibrated sets, or else it is discarded. We repeat this process until having 1000 calibrated sets.

| **Calibration statistics** | **Target range** |
| --- | --- |
| Adult population size  (millions) | 27.354-29.046 |
| HIV prevalence  (% of HIV+ in population) | 17.5%-20.3% |
| HIV incidence  (per 1000 susceptible individuals) | 13.8-20.6 |
| ART coverage  (% of HIV+ on ART) | 25.6%-32.5% |
| % of HIV+ undiagnosed | 45%-55% |

**Table S10 – Calibration statistics and targets (9)**

The calibrated simulations are then continued until 2020 when different TPR-preserving strategies are initiated.

**6. TPR definitions**

In our main analysis, the TPR is defined as follows for a given year :

$$\mathrm{TPR}(k)= \frac{\# of new infections during year k}{\# of new ART initiations during year k}$$

In our secondary analysis, the net TPR is defined as follows for a given year :

$$\mathrm{NetTPR}(k)= \frac{\# of new infections during year k}{difference in \# of individuals on ART between start of year k+1 and start of year k}$$

**7. TPR-preserving strategies**

At the end of 2019, we measure the TPR and then use it as a target for ART scale-up over 15 years (2020-2034). Over 1000 calibrated simulations, we obtain TPR values in the range 0.65-1.25. We select three simulations for further analysis: all have the same HIV incidence of 14 yearly new infections per 1000 HIV- in 2019, but one has high TPR (1.24), one has balanced TPR (1), and one has low TPR (0.84).

In the period 2020-2034, we use different TPR-preserving interventions to match the value of TPR measured in 2019. Each year, this is achieved by moving a number $M$ of infected individuals to ART proportionally to the number of new infections that occurred during the previous year (with the proportion chosen to match 2019’s TPR). To achieve this, we implement the eight strategies described in Table S11 (in all cases, individuals move to ART at once at the beginning of the year, every year).

The “CD4<XX first” strategies represent an optimistic model of intervention where undiagnosed HIV+ individuals are quickly identified and moved to treatment. The “CD4<XX priority” strategies, more realistically, model an intervention where base-case ART initiation rates are maintained and supplemented, preferentially by diagnosed HIV+ individuals not on treatment.

| **TPR strategy** | **Description** |
| --- | --- |
| CD4 <200 first | Individuals start ART from $I_{5}^{D}$ first, then from $I_{5}$ if more are needed, then from $I_{4}^{D}$ if more are needed, then from $I_{4}$ if more are needed, until reaching $M$ individuals. The possibility of passing individuals from other compartments is kept open, but in all simulations, there are enough individuals in these four compartments to match 2019’s TPR value over 2020-2034. |
| CD4 <200 priority | Diagnosed individuals ($I_{k}^{D}$ for $k=2, 3, 4, 5$) initiate ART following the base-case yearly rates. Additional individuals are selected from diagnosed and undiagnosed compartments, following the order in CD4 <200 first strategy. |
| CD4 <350 first | Individuals start ART from $I_{5}^{D}$ and $I_{4}^{D}$ proportionally to the count of these two compartments ($M\frac{I_{5}^{D}}{I_{4}^{D}+I_{5}^{D}}$ from $I_{5}^{D}$ and $M\frac{I_{4}^{D}}{I_{4}^{D}+I_{5}^{D}}$ from $I_{4}^{D}$). If there are not enough individuals in both compartments, more are selected from $I_{4}$ and $I_{5}$ to meet the required numbers. |
| CD4 <350 priority | Diagnosed individuals ($I_{k}^{D}$ for $k=2, 3, 4, 5$) initiate ART following the base-case yearly rates. Additional individuals are selected from $I_{5}^{D}$ and $I_{4}^{D}$ proportionally to the base-case yearly rates for these two compartments. |
| CD4 <500 first | Individuals moved to ART from $I_{5}^{D}$, $I_{4}^{D}$ and $I_{3}^{D}$proportionally to the count of these three compartments ($M\frac{I_{5}^{D}}{I_{3}^{D}+I_{4}^{D}+I_{5}^{D}}$ from $I_{5}^{D}$, $M\frac{I_{4}^{D}}{I_{3}^{D}+I_{4}^{D}+I_{5}^{D}}$ from $I_{4}^{D}$ and $M\frac{I_{3}^{D}}{I_{3}^{D}+I_{4}^{D}+I_{5}^{D}}$ from $I_{3}^{D}$). If there are not enough individuals in these compartments, more are selected from $I_{3},$ $I_{4}$ and $I_{5}$ to meet the required numbers. |
| CD4 <500 priority | Diagnosed individuals ($I_{k}^{D}$ for $k=2, 3, 4, 5$) initiate ART following the base-case yearly rates. Additional individuals are selected from $I_{5}^{D}$, $I_{4}^{D}$ and $I_{3}^{D}$ proportionally to the base-case yearly rates for these three compartments. |
| Universal first | Individuals moved to ART from $I_{5}^{D}$, $I_{4}^{D}$, $I_{3}^{D}$ and $I_{2}^{D}$proportionally to the count of these three compartments ($M\frac{I_{5}^{D}}{I_{2}^{D}+I_{3}^{D}+I_{4}^{D}+I_{5}^{D}}$ from $I_{5}^{D}$, $M\frac{I_{4}^{D}}{I_{2}^{D}+I_{3}^{D}+I_{4}^{D}+I_{5}^{D}}$ from $I_{4}^{D}$, $M\frac{I_{3}^{D}}{I_{2}^{D}+I_{3}^{D}+I_{4}^{D}+I_{5}^{D}}$ from $I_{3}^{D}$ and $M\frac{I_{2}^{D}}{I_{2}^{D}+I_{3}^{D}+I_{4}^{D}+I_{5}^{D}}$ from $I_{2}^{D}$). If there are not enough individuals in these compartments, more are selected from $I_{2}$, $I_{3},$ $I_{4}$ and $I_{5}$ to meet the required numbers. |
| Universal priority | Diagnosed individuals ($I_{k}^{D}$ for $k=2, 3, 4, 5$) initiate ART following the base-case yearly rates. Additional individuals are selected from $I_{5}^{D}$, $I_{4}^{D}$, $I_{3}^{D}$ and $I_{2}^{D}$ proportionally to the base-case yearly rates for these four compartments. |

**Table S11 – TPR-preserving strategies**

**8. Additional results**

Model calibration.


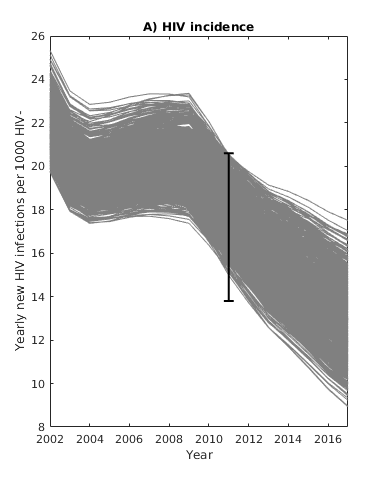

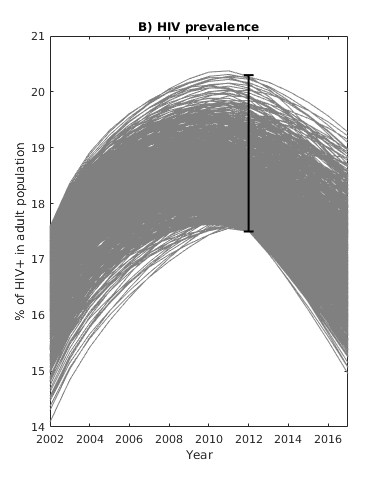


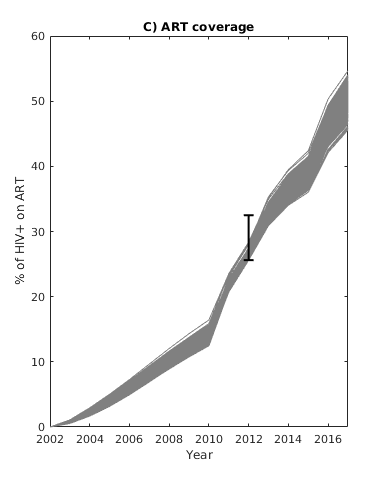

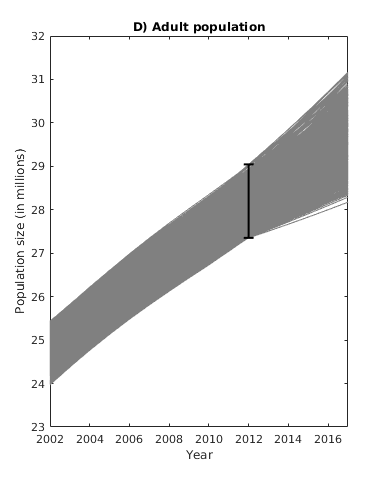


**Figure S1. 1000 calibrated simulations.**

Results for 1000 calibrated simulations.

Figure S2 shows scatter plots of TPR values and HIV incidence reduction for the year 2034 (end of intervention). Tables S12-S20 describe the distribution of ART initiations per CD4 stage.


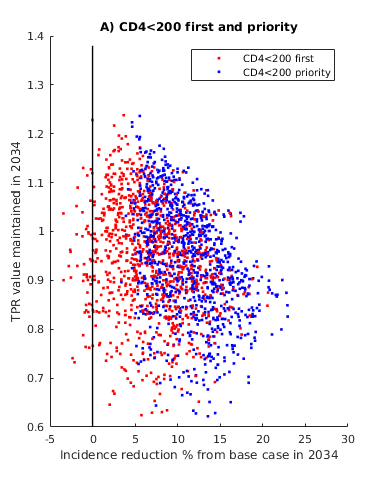

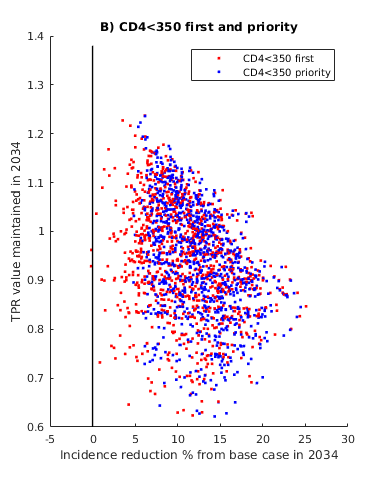

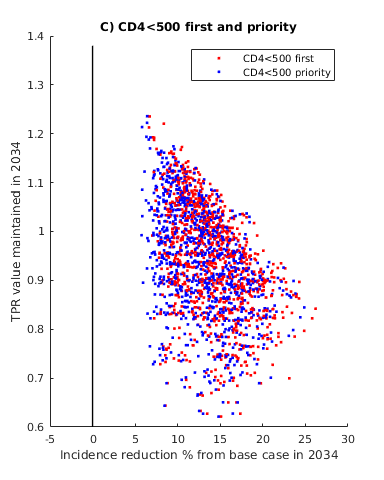

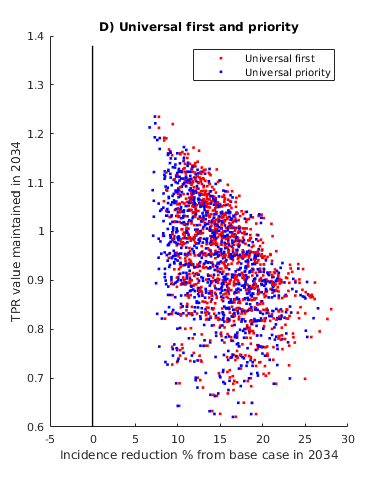


**Figure S2. TPR vs. HIV incidence reduction (1000 simulations).**

| Year \ CD4 | >500 | 350-500 | 200-350 | <200 |
| --- | --- | --- | --- | --- |
| 2020 | 3.87-8.7 | 19.4-33.5 | 24.8-33.5 | 27-47.9 |
| 2027 | 4.06-9.38 | 19-34.6 | 23.9-33.5 | 25.9-48.5 |
| 2034 | 4.24-9.5 | 19.5-35 | 23.9-33.6 | 25.3-47.7 |

**Table S12. Base case.** Distribution (%) of new ART initiations by CD4 stage (range over 1000 simulations).

| Year \ CD4 | >500 | 350-500 | 200-350 | <200 |
| --- | --- | --- | --- | --- |
| 2020 | 0-0 | 0-0 | 0-39.5 | 60.5-100 |
| 2027 | 0-0 | 0-0 | 30.7-67.7 | 32.3-69.3 |
| 2034 | 0-0 | 0-0 | 34.6-71.9 | 28.1-65.4 |

**Table S13. CD4 <200 first.** Distribution (%) of new ART initiations by CD4 stage (range over 1000 simulations).

| Year \ CD4 | >500 | 350-500 | 200-350 | <200 |
| --- | --- | --- | --- | --- |
| 2020 | 2.77-5.77 | 14.3-23.7 | 17-23.7 | 47.7-63.4 |
| 2027 | 3.51-7.82 | 17-30.4 | 20.6-30.7 | 34.8-54.2 |
| 2034 | 3.5-7.82 | 16.9-30.3 | 19.8-29.9 | 35.5-55.3 |

**Table S14. CD4 <200 priority.** Distribution (%) of new ART initiations by CD4 stage (range over 1000 simulations).

| Year \ CD4 | >500 | 350-500 | 200-350 | <200 |
| --- | --- | --- | --- | --- |
| 2020 | 0-0 | 0-0 | 54.3-77.2 | 22.8-45.7 |
| 2027 | 0-0 | 0-0 | 52.2-77.9 | 22.1-47.8 |
| 2034 | 0-0 | 0-0 | 53.4-80.4 | 19.6-46.6 |

**Table S15. CD4 <350 first.** Distribution (%) of new ART initiations by CD4 stage (range over 1000 simulations).

| Year \ CD4 | >500 | 350-500 | 200-350 | <200 |
| --- | --- | --- | --- | --- |
| 2020 | 2.77-5.77 | 14.3-23.7 | 32.2-38.8 | 35.3-47.5 |
| 2027 | 3.5-7.82 | 17.1-30.5 | 25.4-34.6 | 30.4-50.2 |
| 2034 | 3.5-7.82 | 16.9-30.3 | 25.1-34.6 | 30.8-50.5 |

**Table S16. CD4 <350 priority.** Distribution (%) of new ART initiations by CD4 stage (range over 1000 simulations).

| Year \ CD4 | >500 | 350-500 | 200-350 | <200 |
| --- | --- | --- | --- | --- |
| 2020 | 0-0 | 30.2-44.6 | 35.6-47 | 12.8-30.2 |
| 2027 | 0-0 | 26.2-49 | 24.2-37.2 | 19.6-42.7 |
| 2034 | 0-0 | 26.5-51.1 | 24-38 | 18.1-42 |

**Table S17. CD4 <500 first.** Distribution (%) of new ART initiations by CD4 stage (range over 1000 simulations).

| Year \ CD4 | >500 | 350-500 | 200-350 | <200 |
| --- | --- | --- | --- | --- |
| 2020 | 2.77-5.77 | 24.9-33.5 | 28.5-34.2 | 29.1-40.5 |
| 2027 | 3.51-7.83 | 20-34.5 | 23-32.6 | 28.9-48.7 |
| 2034 | 3.5-7.82 | 20.4-34.9 | 22.3-32.2 | 29.1-48.8 |

**Table S18. CD4 <500 priority.** Distribution (%) of new ART initiations by CD4 stage (range over 1000 simulations).

| Year \ CD4 | >500 | 350-500 | 200-350 | <200 |
| --- | --- | --- | --- | --- |
| 2020 | 5.44-10.3 | 28.2-40.2 | 32.9-44.3 | 11.7-28.3 |
| 2027 | 5.61-13.1 | 20.9-37.7 | 23.2-36 | 19.8-42.7 |
| 2034 | 5.92-13.9 | 21.1-38.9 | 22.9-36.7 | 18.2-42.1 |

**Table S19. Universal first.** Distribution (%) of new ART initiations by CD4 stage (range over 1000 simulations).

| Year \ CD4 | >500 | 350-500 | 200-350 | <200 |
| --- | --- | --- | --- | --- |
| 2020 | 11.2-14.5 | 22.8-31.1 | 26.1-31.6 | 25.4-36.7 |
| 2027 | 7.48-14.7 | 17.8-31.1 | 21.7-31.7 | 27.3-47 |
| 2034 | 8.4-16.7 | 17.9-31.2 | 20.6-31.2 | 27-46.4 |

**Table S20. Universal priority.** Distribution (%) of new ART initiations by CD4 stage (range over 1000 simulations).

Temporal dynamic of the epidemic with different TPR values maintained


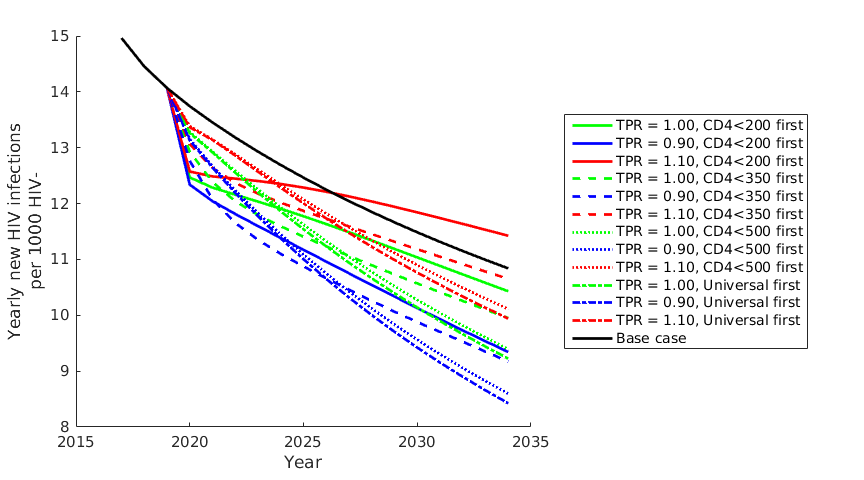


**Figure S3. TPR-preserving strategies under identical epidemic conditions:** Comparison of HIV incidence curves under different ART expansion strategies maintaining TPR at 0.9, 1 or 1.1 after 2019.

Temporal dynamic of the epidemic with modified ART dropout rates


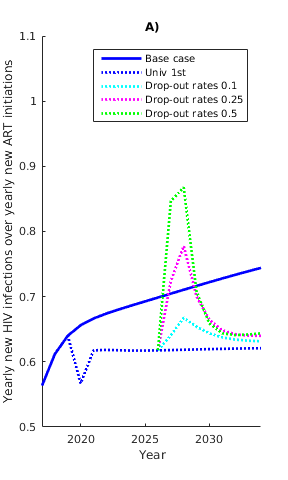

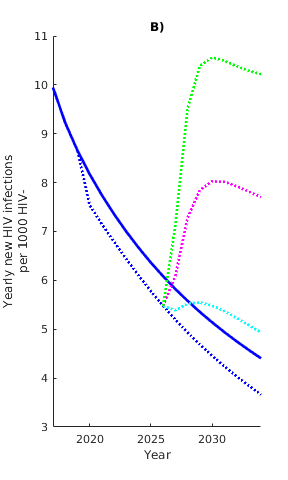

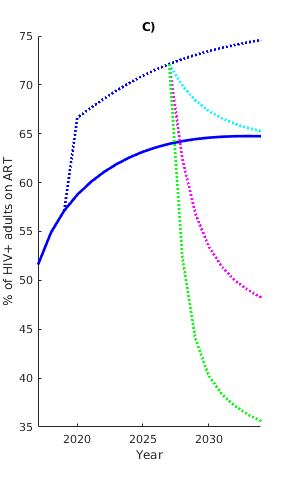


**Figure S4. “Universal first” strategy: effect of augmentation of ART dropout rates on** A) TPR; B) HIV incidence and C) ART coverage. The TPR value rises in 2027 because i) the numerator (new infections in 2027) increases due to ART dropout, and ii) the denominator (new ART initiations in 2027) does not increase because it is based on infections that occurred during the year 2026. The denominator catches up with new infections afterwards, and the TPR value falls back near the pre-2027 level.

Simulations with alternative TPR definition

**
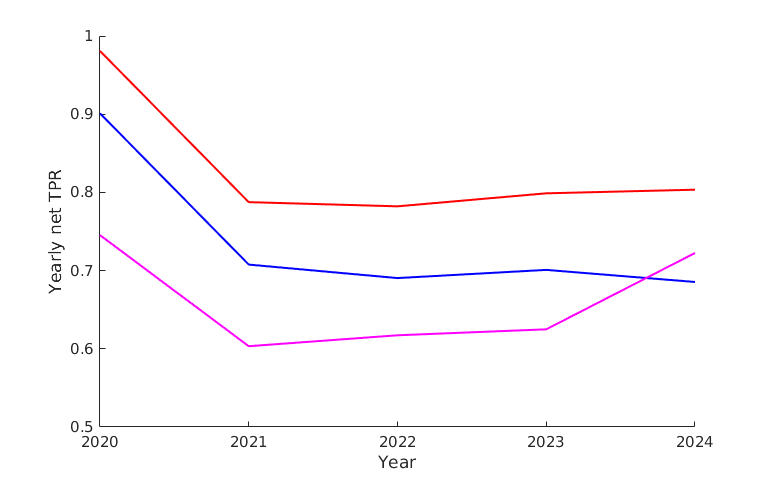
**

**Figure S5. Net TPR:** Fluctuation of net TPR value in time (three selected simulations from the 1000 calibrated simulations, under “universal first” strategy).

**
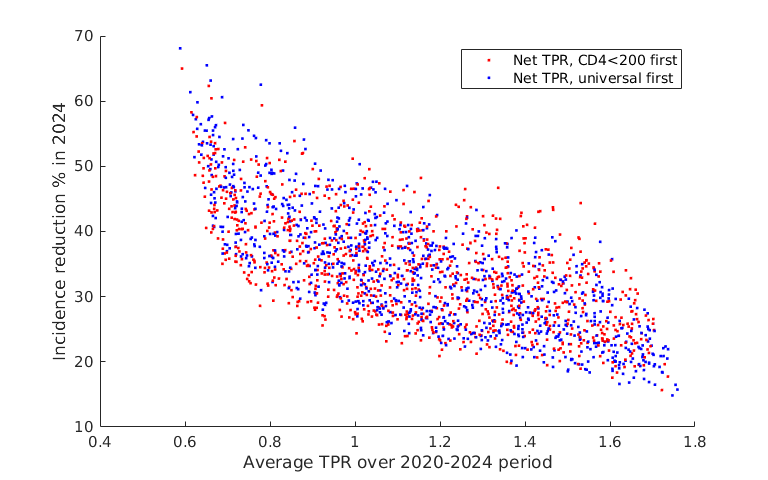
**

**Figure S6. Net TPR:** Comparison of “CD4<200 first” and “universal first” strategies.

**9. Supplementary information references**

1. The World Bank. Health Nutrition and Population Statistics (2015) Female population 15-19, 20-24, 25-29, 30-34, 35-39, 40-44, 45-49 and Male population 15-19, 20-24, 25-29, 30-34, 35-39, 40-44, 45-49 in South Africa 1970-2014.

2. Wawer MJ*, et al.* (2005) Rates of HIV-1 transmission per coital act, by stage of HIV-1 infection, in Rakai, Uganda. *Journal of Infectious Diseases* 191(9):1403-1409.

3. Todd J*, et al.* (2007) Time from HIV seroconversion to death: a collaborative analysis of eight studies in six low and middle-income countries before highly active antiretroviral therapy. *AIDS* 21:S55-S63.

4. Lodi S, et al. (2011) Time from Human Immunodeficiency Virus seroconversion to reaching CD4+ cell count thresholds < 200, < 350, and < 500 cells/mm^3^ : Assessment of need following changes in treatment guidelines. *Clinical Infectious Diseases* 53:817–825.

5. Cohen MS*, et al.* (2011) Prevention of HIV-1 Infection with Early Antiretroviral Therapy. *New England Journal Of Medicine* 365(6):493-505.

6. Eaton JW*, et al.* (2012) HIV Treatment as Prevention: Systematic Comparison of Mathematical Models of the Potential Impact of Antiretroviral Therapy on HIV Incidence in South Africa. *PLoS Med* 9(7):e1001245-.

7. Beksinska ME, *et al.* (2012) Progress and challenges to male and female condom use in South Africa. *Sexual Health* 9:51-58.

8. Davis KR, Weller SC (1999) The effectiveness of condoms in reducing heterosexual transmission of HIV. *Family Planning Perspectives* 31:272-279.

9. Shisana O, *et al.* (2014) *South African National HIV Prevalence, Incidence and Behaviour Survey, 2012* (HSRC Press, Cape Town, South Africa).

10. Johnson LF (2012) Access to antiretroviral treatment in South Africa, 2004-2011. Southern African Journal of HIV Medecine 13(1):22-27.
